# Application of Large Language Models in Data Analysis and Medical Education for Assisted Reproductive Technology: Comparative Study

**DOI:** 10.2196/70107

**Published:** 2025-10-01

**Authors:** Noriyuki Okuyama, Mika Ishii, Yuriko Fukuoka, Hiromitsu Hattori, Yuta Kasahara, Tai Toshihiro, Koki Yoshinaga, Tomoko Hashimoto, Koichi Kyono

**Affiliations:** 1 Kyono ART Clinic Takanawa, Takanawa Court 5F, 3-13-1 Takanawa, Minato-ku, Tokyo, 108-0074, Japan, 81 364084708, 81 364084702; 2 Kyono ART Clinic Sendai, Sendai, Japan; 3 Kyono ART Clinic Morioka, Morioka, Japan

**Keywords:** artificial intelligence, large language model, data analysis, education, infertility

## Abstract

**Background:**

Recent studies have demonstrated that large language models exhibit exceptional performance in medical examinations. However, there is a lack of reports assessing their capabilities in specific domains or their application in practical data analysis using code interpreters. Furthermore, comparative analyses across different large language models have not been extensively conducted.

**Objective:**

The purpose of this study was to evaluate whether advanced artificial intelligence (AI) models can analyze data from template-based input and demonstrate basic knowledge of reproductive medicine. Four AI models (GPT-4, GPT-4o, Claude 3.5 Sonnet, and Gemini Pro 1.5) were evaluated for their data analytical capabilities through numerical calculations and graph rendering. Their knowledge of infertility treatment was assessed using 10 examination questions developed by experts.

**Methods:**

First, we uploaded data to the AI models and furnished instruction templates using the chat interface. The study investigated whether the AI models could perform pregnancy rate analysis and graph rendering, based on blastocyst grades according to Gardner criteria. Second, we assessed model diagnostic capabilities based on specialized knowledge. This evaluation used 10 questions derived from the Japanese Fertility Specialist Examination and the Embryologist Certification Exam, along with chromosome imaging. These materials were curated under the supervision of certified embryologists and fertility specialists. All procedures were repeated 10 times per AI model.

**Results:**

GPT-4o achieved grade A output (defined as achieving the objective with a single output attempt) in 9 out of 10 trials, outperforming GPT-4, which achieved grade A in 7 out of 10. The average processing times for data analysis were 26.8 (SD 3.7) seconds for GPT-4o and 36.7 (SD 3) seconds for GPT-4, whereas Claude failed in all 10 attempts. Gemini achieved an average processing time of 23 (SD 3) seconds and received grade A in 6 out of 10 trials, though occasional manual corrections were needed. Embryologists required an average of 358.3 (SD 9.7) seconds for the same tasks. In the knowledge-based assessment, GPT-4o, Claude, and Gemini achieved perfect scores (9/9) on multiple-choice questions, while GPT-4 showed a 60% (6/10) success rate on 1 question. None of the AI models could reliably diagnose chromosomal abnormalities from karyotype images, with the highest image diagnostic accuracy being 70% (7/10) for Claude and Gemini.

**Conclusions:**

This rapid processing demonstrates the potential for these AI models to significantly expedite data-intensive tasks in clinical settings. This performance underscores their potential utility as educational tools or decision support systems in reproductive medicine. However, none of the models were able to accurately interpret and diagnose using medical images.

## Introduction

The term “artificial intelligence” (AI) was first defined at the Dartmouth Summer Research Project on Artificial Intelligence in 1956. Over half a century after this seminal conference, advances in computational power and exponential growth in data available for machine learning have ushered in the third AI boom, commencing after 2010 [1]. A particularly significant milestone was reached in November 2022 with the release of ChatGPT (OpenAI), a large language model (LLM) developed by OpenAI. This model had an unprecedented impact, amassing a global user base of 100 million within just 2 months of its public launch. The field has since progressed beyond generative AI to what is now termed multimodal AI, capable of integrating and drawing inferences from multiple input modalities, including image, voice, and text. This evolution represents a significant leap in AI capabilities and potential applications. As AI continues to reshape business models and medical education, there are growing expectations for its role in enhancing organizational performance and driving transformative change in various sectors, including health care [2-4]. Implications of these advances for medical practice, research, and health care delivery systems are profound and call for both careful and bold decision-making to drive further progress.

LLMs have achieved substantial improvements through self-attention mechanisms and transformer architectures. Fine-tuning enables domain-specific customization, but it requires high computational costs and expert oversight. To promote health care digital transformation, more accessible methods are needed. Cloud-based code interpreter functionality offers a solution by enabling LLMs to automatically generate and execute code from natural language instructions. This capability supports diverse file formats (CSV, XLSX, DOCX, TXT, and PDF) and performs numerical analysis and visualization online. In medical research, this technology can significantly enhance efficiency through rapid dataset processing, statistical analysis, and graph generation [5]. Another important application is the use of these models as chatbot tools. While there have been initial reports on the use of early GPT models in infertility treatment, there is a notable lack of literature evaluating practical support capabilities and response quality of updated LLMs in this field [6,7]. Updating the current assessment of AI capabilities could provide clearer insights into the challenges and opportunities for AI use in medical education and patient counseling in reproductive medicine. This evaluation is essential to identify specific areas in which AI can effectively complement human expertise to improve patient care in fertility treatment contexts.

The field of infertility treatment has seen a growing number of reports on AI and machine learning applications, including survival rate prediction based on gradient-based localization of blastocyst images, iDAscore, TESE sperm search, and detection of oocyte fragility during intracytoplasmic sperm injection, among other topics [8-11]. However, implementing these advanced technologies in individual clinics often requires specific equipment, software, and specialized personnel, which can be challenging. Additionally, while real-time capabilities for quality control and treatment management in fertility care are of paramount importance, there remains a significant disparity in computational infrastructure capacity across health care institutions. Notably, many medical facilities, particularly smaller clinics, lack the requisite computing infrastructure necessary for executing sophisticated AI or machine learning models. This infrastructural limitation constitutes a substantial barrier to the widespread implementation of advanced data-driven approaches in reproductive medicine [12]. Recognizing this, we hypothesized that selecting and using more accessible AI tools could promote digital transformation among infertility treatment professionals. This study evaluated 4 accessible AI models for infertility treatment applications, assessing their data processing capabilities and knowledge performance. These 4 AI models—GPT-4 (OpenAI), GPT-4o (OpenAI), Claude 3.5 Sonnet (Anthropic), and Gemini Pro 1.5 (Google)—were selected because they (1) are pretrained on trillion-token-scale corpora, (2) can be accessed immediately through simple account registration without local computational resources, and (3) therefore represent the most widely reproducible options for clinicians and educators seeking to leverage cutting-edge LLM technology in real-world settings.

This assessment comprised 2 key components. First, the ability of these models to process and analyze clinical data using the code interpreter feature, specifically calculating and visualizing pregnancy rates stratified by blastocyst trophectoderm grades, and second, their performance on a comprehensive knowledge test in reproductive medicine. This test consisted of 10 multiple-choice questions, each with 5 options, covering various aspects of assisted reproductive technology (ART). The questions were meticulously curated and validated by a multidisciplinary panel of experts, including certified clinical embryologists, gynecologists, and urologists specializing in reproductive medicine. By examining these 2 crucial areas, the study assessed the current capabilities and limitations of AI in processing complex reproductive medical data, as well as its potential role in medical education in this specialized field.

## Methods

### AI Models and the Risk of Personal Information Leakage

This study used GPT-4, GPT-4o, Claude 3.5 Sonnet, and Gemini Pro 1.5 for data analysis. The dataset was structured with analysis items in the first column and corresponding data in rows. Data formatting was consistent, avoiding mixed character types and unnecessary spaces. Each cell contained a single data point. All identifying information for patients and staff was removed from datasets before analysis. The study did not use ChatGPT’s memory feature or Gemini’s extended functionalities. No prompt engineering techniques were used to optimize outputs. Null data were included, and each dataset remained in the original format as output from the database software. Additionally, no prompt engineering techniques or fine-tuning were used to optimize the outputs. Generative AI tools were not used for the generation of references or the writing of this manuscript. All citations were verified manually, and all textual content was written and reviewed by the authors. However, AI tools were used for grammar checking prior to professional English editing.

### Data Collection

**Figure 1.**
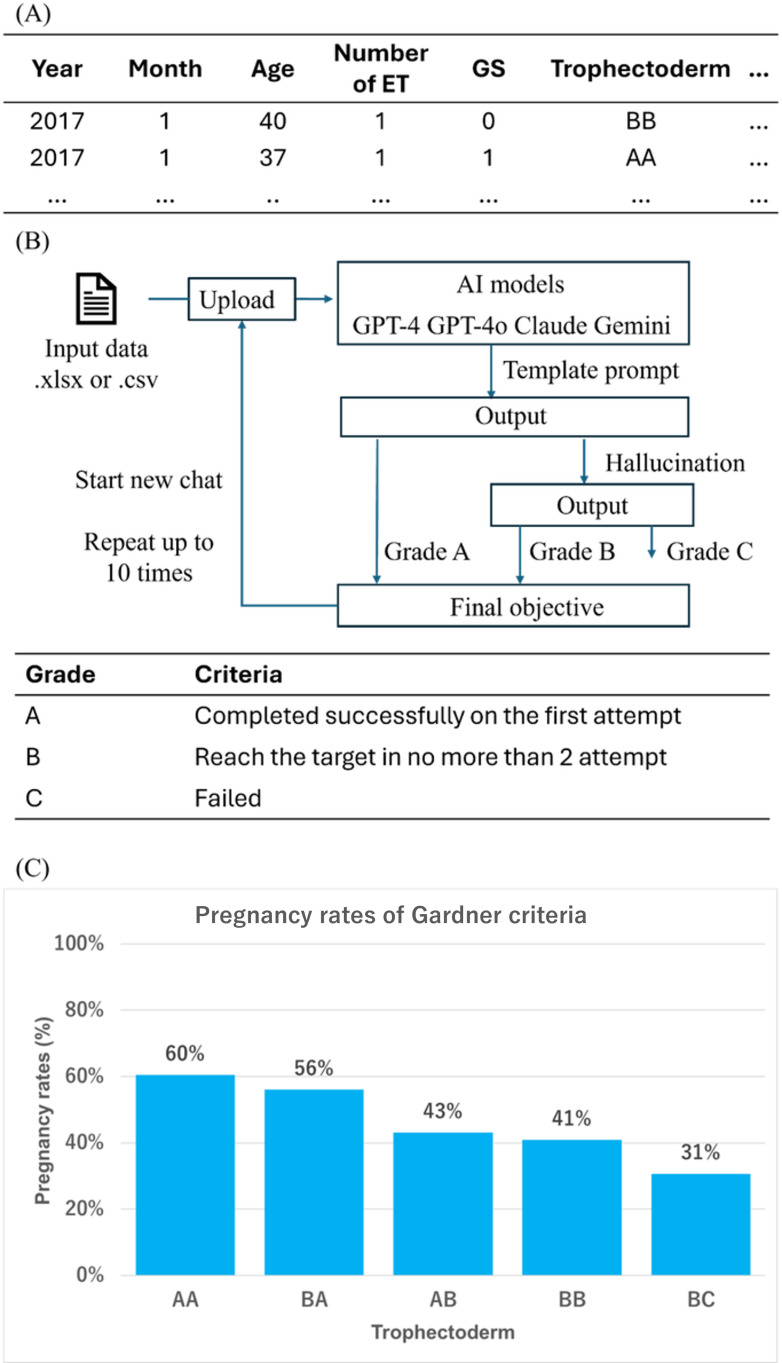
Data analysis procedure and evaluation framework for large language models in assisted reproductive technology clinical data processing. (**A**) Sample dataset structure showing patient treatment data from frozen-thawed embryo transfer cycles (January 2017-July 2024; 5361 cycles from 2276 patients) formatted for artificial intelligence (AI) model input with variables including patient age, embryo quality, and pregnancy outcomes. (**B**) Study workflow showing systematic evaluation protocol where 4 AI models (GPT-4, GPT-4o, Claude 3.5 Sonnet, and Gemini 1.5 Pro) were tested 10 times each using standardized template prompts, with performance graded as A, B, or C. (**C**) Target visualization output showing pregnancy rates stratified by Gardner criteria trophectoderm grades (AA, BA, AB, BB, and BC) from clinic data. ET: embryo transfer; GS: gestational sac.

This study analyzed pregnancy rates by embryo grade in frozen-thawed embryo transfer cycles using data from January 2017 to July 2024, encompassing 5361 cycles from 2276 patients. The dataset was prepared as a 362 KB Microsoft Excel file with 32 columns and 5321 rows. The dataset was initially generated using Claris FileMaker, a relational database platform, and then exported into Microsoft Excel format for use in AI models. In the exported file, the first row contained the names of analysis variables (eg, patient age, embryo quality, and pregnancy outcome), while subsequent rows held corresponding numeric or categorical values for each case. Each cell was carefully formatted to contain only a single, discrete data element—without the use of delimiters, such as slashes or commas—to ensure clarity and prevent parsing errors during AI processing. This standardized structure enabled consistent interpretation across platforms, regardless of differences in input limitations. Claude’s procedure differed slightly due to lower file size limitations. It used a reduced dataset of 5 columns and 3000 rows in CSV format to avoid “length over” errors, while maintaining the same basic operation and template prompts as the other AI models (Figure 1A). In contrast, GPT-4, GPT-4o, and Gemini were evaluated using the full dataset (32 columns and 5321 rows), allowing direct comparison of their performance under uniform data conditions.

### Data Analysis

The methodology involved uploading data to each AI model and applying a standard instruction template (Textbox 1). Performance was rated on a scale “A” for correct output on the first attempt, “B” for correction within 2 attempts, and “C” for errors or requiring 3 or more attempts. This process was repeated 10 times for each LLM, with a new chat initiated after each round to prevent memory influence (Figure 1B). This approach allowed systematic evaluation of AI capabilities in processing and visualizing complex clinical data while accounting for platform-specific constraints. The analysis sought to produce a bar graph with data labels categorized by Gardner criteria (Figure 1C). Compared with embryologists who routinely perform data processing, 3 embryologists carried out calculations under similar conditions using Microsoft Excel.

Textbox 1. **Template prompt of data analysis.**

Pregnancy rates are calculated by “Trophectoderm”.

X-axis is “Trophectoderm” in the order AA, BA, AB, BB, BC

Y-axis is 0-100%.

Please add data labels for each value

Draw a bar graph for the graph.

Graph title is “Pregnancy Rates of Gardner Criteria”

Pregnancy rate definition:

Numerator: “GS” total number

Denominator: “numberofET” number of data entries

Search for "GS" and "numberofET" in the column and calculate

### Assessment of Knowledge of Infertility

**Figure 2.**
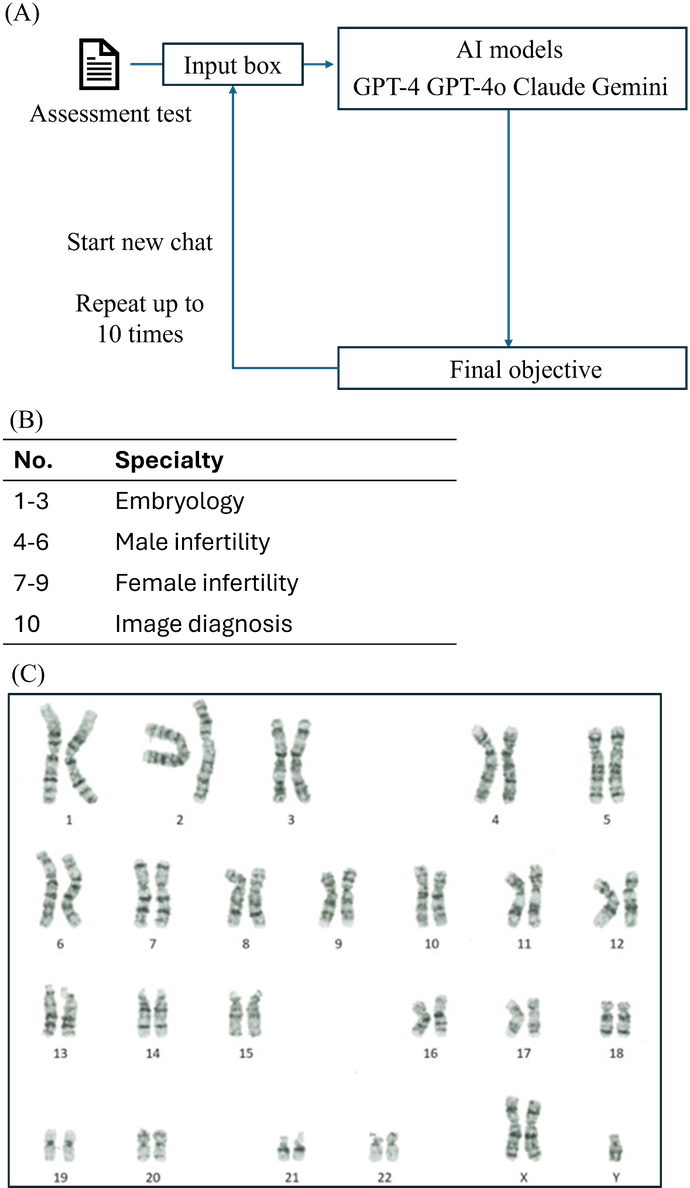
Knowledge assessment framework for large language models in reproductive medicine education. (**A**) Study protocol for evaluating artificial intelligence (AI) model performance on fertility specialist examination questions, with 10 independent trials per model using fresh chat sessions. (**B**) Question distribution showing sources: 3 questions from a senior embryologist, 6 questions from board-certified specialists (Japan Society for Reproductive Medicine gynecology and urology specialist exams, 2016‐2018), and 1 image-based karyotype diagnosis question. (**C**) Karyotype analysis test image (600×450 pixels) used for chromosomal abnormality diagnosis assessment across all AI models.

GPT-4, GPT-4o, Claude, and Gemini used a set of 10 multiple-choice questions with options a-e (Textbox 2). Questions were input into each AI interface, and the process was repeated 10 times, initiating a new chat session for each round to ensure independent responses (Figure 2A). Questions were carefully curated. Overall, 3 questions were prepared by a senior embryologist, while board-certified specialists from the Japan Society for Reproductive Medicine in gynecology and urology each selected 3 questions from the gynecology specialist exam (2016‐2018; Figure 2B). As a final question, the assessment included image-based diagnosis using chromosome diagrams. The image used for analysis was 600×450 pixels (Figure 2C). Gemini occasionally hesitated on highly specialized questions, such as Question 10, sometimes offering only 3 choices or declining to answer with statements like, “I’m a language model and don’t have the capacity to help with that.” In such instances, AI was prompted to provide a definitive choice among the given options.

Textbox 2. **Multiple-choice questions provided to GPT-4, GPT-4o, Claude 3.5 Sonnet, and Gemini Pro 1.5.**

1. Select the correct option regarding the maturation of oocytes before fertilization in chronological order of changes:
  - GV → GVBD → Metaphase I → Anaphase I → Telophase I → Metaphase II
  - GVBD → GV → Metaphase I → Telophase I → Anaphase I → Metaphase II
  - GV → GVBD → Metaphase I → Telophase I → Anaphase I → Metaphase II
  - GVBD → GV → Anaphase I → Metaphase I → Telophase I → Metaphase II
  - GV → GVBD → Anaphase I → Metaphase I → Telophase I → Metaphase II

1. Despite injecting only one sperm into the oocyte cytoplasm during intracytoplasmic sperm injection (ICSI), three pronuclei may sometimes be observed during fertilization confirmation. Which of the following is the most likely cause?
  - Parthenogenesis
  - Polyspermy
  - Retained sperm acrosome
  - Normal fertilization
  - Failure to extrude the second polar body

1. Select the correct statement about glutamine from the following:
  - A low-cytotoxicity buffer used for culture media in air, which does not require CO₂ for buffering capacity
  - An amino acid essential for embryo development but can produce toxic ammonia that affects embryo development; therefore, it is often added to culture media in a stabilized form
  - Added to culture media to prevent the growth of contaminants in case of contamination
  - Has antioxidant, osmotic pressure maintenance, and detoxification effects; also acts as an anti-adhesive agent to prevent embryos from adhering to glass or plastic
  - Forms a chelate with magnesium ions, lowering their concentration, thus preventing abnormal metabolic shifts towards glycolysis and promoting embryo development

1. Which of the following is correct?
  - About 80% of male infertility is caused by varicocele
  - The most common chromosomal abnormality causing male infertility is Klinefelter syndrome
  - ICSI is performed using a method called TESE, which retrieves sperm from the epididymis
  - FSH primarily binds to receptors located in Leydig cells in the testes
  - LH promotes the secretion of Müllerian inhibiting substance (MIS)

1. Which of the following statements about spermatogenesis is incorrect? Choose one.
  - Sertoli cells are stimulated by FSH and are involved in spermatogenesis
  - Leydig cells are stimulated by LH and produce testosterone
  - Sertoli cells respond to androgen and produce factors necessary for spermatogenesis
  - Spermatogonia have androgen receptors and differentiate into sperm cells
  - The hypothalamus-pituitary-gonadal axis plays an important role in spermatogenesis

1. For patients eligible for TESE (testicular sperm extraction), which of the following are predictors of sperm retrieval? Choose two.
  - FSH
  - Testicular volume
  - Complete AZF deletion
  - 46XX male
  - DAZ microdeletion

1. Which conditions have normal ovarian function? Choose two.
  - Turner syndrome
  - Klinefelter syndrome
  - Swyer syndrome
  - Asherman syndrome
  - Mayer-Rokitansky-Küster-Hauser (MRKH) syndrome

1. This question is about the correct statement regarding the luteinization process in humans. The correct answer is:
  - Luteinization is completed within about 2 days after ovulation
  - The mature corpus luteum after luteinization is also called the “corpus rubrum”
  - Angiogenesis is induced during the luteinization phase
  - Granulosa cells release intracellular granules
  - Estrogen production decreases

1. This question is about hormones primarily produced by the adrenal glands in females. The correct answer is:
  - Androstenedione
  - Testosterone
  - Dehydroepiandrosterone sulfate (DHEA-S)
  - Dihydrotestosterone (DHT)
  - Sex hormone-binding globulin (SHBG)

1. Here is a karyotype image. Please select the possible diagnosis based on this image: (Figure 2C)
  - Klinefelter syndrome
  - Turner syndrome
  - Down syndrome
  - Chromosomal translocation
  - Normal

### Ethical Considerations

This is a retrospective study using patient treatment data from January 2017 to July 2024. Informed consent for data analysis and secondary use was obtained from all patients. Patient data were not used in the testing of specialized knowledge. All data used in this study were deidentified to ensure the privacy and confidentiality of human candidates. This study was conducted with the approval of the institutional ethics committee (5805‐240224) and did not receive any special funding.

## Results

### Data Analysis

GPT-4 achieved grade A performance in 7 out of 10 attempts, while GPT-4o excelled with 9 grade A performances. GPT-4o also demonstrated superior processing speed, averaging 26.8 (SD 3.7) seconds compared to GPT-4’s 36.7 (SD 3) seconds. GPT-4 encountered issues with column recognition in 3 instances, resolved by requesting another search (Table 1). GPT-4o had minor issues with Y-axis scaling in graph outputs, defaulting to 0%‐60% instead of 0%‐100%, as instructed, but this was easily corrected. Postcorrection response times further highlighted GPT-4o’s efficiency (41 s vs GPT-4’s 90.7, SD 5.7 s). Claude consistently performed at grade C, failing to process the entire dataset and producing incomplete outputs. Despite attempts to specify row numbers and implement programs to read to the final row, Claude was unable to produce the desired numerical results or graphical representations. Gemini prompted users to select a graphing tool or library (such as Microsoft Excel, Google Sheets, or Python’s Matplotlib) for visualization. It also occasionally included null values in graph representations without exclusion. When a staff member with the fastest data processing skills performed numerical calculations and generated graphical charts using the same datasets, approximately 7 minutes were required. Furthermore, values evaluated as “A” or “B” by the LLM were consistent with those verified by the staff members.

**Table 1.** Performance comparison of multiple large language models and human embryologists in clinical data processing for assisted reproductive technology. All procedures were repeated 10 times per artificial intelligence model and 3 times for embryologists.

Variables	GPT-4 (n=10)	GPT-4o (n=10)	Claude 3.5 Sonnet (n=10)	Gemini 1.5 Pro (n=10)	Embryologist (n=3)
Output grade, n (%)					
Grade A	7 (70)	9 (90)	0 (0)	6 (60)	3 (100)
Grade B	3 (30)	1 (10)	0 (0)	4 (40)	0 (0)
Grade C	0 (0)	0 (0)	10 (100)	0 (0)	0 (0)
Calculation time (s), mean (SD)					
Grade A	36.7 (3)	26.8 (3.7)	N/A^a^	23 (3)	358.3 (9.7)
Grade B	90.7 (5.7)	41(N/A)	N/A	48.5 (3)	N/A
Type of hallucinations, n (%)					
Search error	3 (30)	0 (0)	0 (0)	0 (0)	0 (0)
Calculation error	0 (0)	0 (0)	10 (100)	0 (0)	0 (0)
Visualization error	0 (0)	1 (10)	0 (0)	4 (40)	0 (0)

a N/A: not applicable.

### Assessment of Knowledge

The knowledge evaluation was conducted independently from the data processing assessment. This analysis of AI performance on medical questions reveals distinct patterns among models. For questions 1‐9, GPT-4 showed high accuracy, with a single inconsistency on Question 5, selecting option “c” incorrectly 4 times, resulting in a 60% (6/10) success rate for that question. GPT-4o, Claude, and Gemini, however, achieved perfect scores on these initial 9 questions. Question 10, involving complex medical interpretation, proved challenging for all models (Table 2). GPT-4 correctly identified the diagnosis in 2 instances; however, it incorrectly diagnosed “Down syndrome” on 2 occasions and “Normal” on 6 occasions. GPT-4o failed to select the correct diagnosis and instead produced outputs of “Down syndrome” and “Chromosomal translocation.” Responses from both GPT models exhibited a high degree of randomness. Claude accurately identified the correct diagnosis in 7 instances, but misclassified 3 cases as “Normal.” Similarly, Gemini provided the correct diagnosis 7 times, while incorrectly diagnosing “Down syndrome” and “Normal” in 3 instances. No occurrences of Turner syndrome were observed in outputs from any of the LLMs.

**Table 2.** Knowledge assessment results for 4 large language models on 10 reproductive medicine specialist examination questions, showing correct answers out of 10 independent trials per model. Overall, 9 questions are multiple-choice (questions 1-9) and 1 is karyotype image diagnosis (question 10).

Question number	GPT-4	GPT-4o	Claude 3.5 Sonnet	Gemini 1.5 Pro
1	10	10	10	10
2	10	10	10	10
3	10	10	10	10
4	10	10	10	10
5	6	10	10	10
6	10	10	10	10
7	10	10	10	10
8	10	10	10	10
9	10	10	10	10
10	2	0	7	7

## Discussion

### Principal Findings

This study evaluated the abilities of 4 major LLMs in clinical data analysis and knowledge assessment in ART. For data analysis tasks involving pregnancy rate calculations stratified by blastocyst grades, human embryologists achieved the highest accuracy with no errors across all trials, though they required significantly more time to complete the task. Among the AI models, GPT-4o demonstrated the best balance of speed and accuracy, while GPT-4 showed intermediate performance with occasional column recognition errors. Gemini exhibited the fastest processing speed but lacked precision, often requiring manual corrections for visualization issues. Notably, Claude 3.5 Sonnet consistently failed to complete the data analysis tasks despite multiple attempts with modified datasets.

All 4 models achieved perfect scores on reproductive medicine knowledge questions derived from specialist certification examinations, demonstrating comprehensive theoretical understanding. However, none of them could reliably interpret chromosomal karyotype images, revealing a critical gap between theoretical knowledge and visual diagnostic capabilities. These findings suggest that LLMs can serve as valuable tools for accelerating routine data processing in clinical settings and as educational resources for foundational learning in reproductive medicine, while they emphasize the continued necessity of human expertise for complex diagnostic tasks.

### Data Analysis

Infertility treatment strives to achieve successful patient pregnancies; thus, reviewing treatment outcomes and establishing key performance indicators are crucial [13]. With the increasing availability of large datasets, there are high expectations for machine learning and AI advances to revolutionize health care. However, actual applications of data-driven medicine remain relatively limited at present [12]. This study revealed substantial performance differences among LLMs in clinical data processing (Table 1). While the specific architectural differences and training datasets of each model remain proprietary, the superior performance of GPT-4o likely reflects optimizations in its code interpreter functionality and enhanced context-handling capabilities. The complete failure of Claude, despite its large context window, suggests that file processing and numerical computation capabilities vary significantly across platforms, regardless of their general language understanding abilities. We calculated pregnancy rates for frozen-thawed embryo transfers categorized by Gardner criteria. GPT-4o demonstrated strong proficiency in both calculations and data retrieval, completing these tasks in an average of 26.8 (SD 3.7) seconds with 90% (9/10) accuracy. Similarly, Gemini 1.5 Pro exhibited rapid computational speed (mean 23, SD 3 s), but it occasionally included extraneous elements beyond the specified instructions. Claude 3.5 Sonnet, despite being provided with a truncated dataset due to upload constraints, struggled to produce the desired output. In contrast, embryologists performed the calculations without error but required an average of 358.3 (SD 9.7) seconds—significantly slower than the LLMs. Previous studies have explored the capabilities of LLMs in medical contexts, including scientific writing with simulated data [5] and the broader potential of AI to transform infertility care. However, real-world applications of data-driven approaches in reproductive medicine remain relatively limited, despite their transformative promise [12]. The performance differences observed, particularly Claude’s inability to process clinical datasets and Gemini’s handling of null values, highlight the practical challenges of implementing these technologies. While previous research has emphasized the need for advanced systems to enhance ART outcomes and support clinical decision-making, our findings offer concrete insights into implementation barriers in actual practice. GPT-4o reduced processing time from approximately 6 minutes for human embryologists to less than 30 seconds, representing more than a simple efficiency improvement. This acceleration enables embryologists to redirect their expertise toward critical hands-on procedures such as intracytoplasmic sperm injection, vitrification, and embryo assessment, rather than spending valuable time on routine data analysis. Looking toward future implementations, this rapid processing capability could potentially enable continuous quality monitoring systems. As LLM integration with laboratory information management systems advances, clinics may be able to implement continuous background analysis of treatment outcomes, thus enabling rapid detection of deviations from established benchmarks.

In our dataset, we avoided the simultaneous use of abbreviations like “GS” and “FHB” to reduce hallucination risks. Our experience suggests that using unabbreviated terms without spaces, for example, “GestationalSac” and “FetalHeartbeat,” and avoiding similar alphabetic sequences can yield more accurate results. Although “in-context learning” techniques—such as background priming, role assignment, iterative correction, rule enforcement, and self-reflection—can enhance LLM performance [14,15], these techniques were intentionally not applied in this study to assess baseline output. Additionally, the prompts were not optimized for individual models. Nevertheless, the observed variation in output suggests that model-specific prompt engineering may significantly influence performance and improve alignment with clinical datasets. LLMs can perform medical data analysis substantially faster than manual methods, suggesting their potential to address workforce shortages and reduce costs in implementing data-driven health care.

### Assessment of Knowledge

This study evaluated the performance of LLMs in reproductive medicine using a specialized set of 10 questions developed under supervision by fertility specialists and certified embryologists. These results demonstrated high accuracy across most models, with GPT-4o, Claude 3.5 Sonnet, and Gemini 1.5 Pro achieving perfect scores. These results demonstrated high accuracy across most models, with GPT-4o, Claude 3.5 Sonnet, and Gemini 1.5 Pro all achieving perfect scores. GPT-4 showed some errors, particularly with Question 5, which focused on spermatogenesis (Table 2). These findings align with previous reports that LLMs perform well in medical examinations. For example, GPT-4 achieved an accuracy of 80%‐100% on the United States Medical Licensing Examination and an average score of 76.3% over 3 attempts on the United Kingdom Medical Licensing Assessment. In Japan, GPT-4 passed the national medical licensing exams from 2018 to 2023, though limitations were noted in responses related to cultural context and national guidelines [16-18]. However, our study identified a limitation in image interpretation. Although the models demonstrated an understanding of chromosomal diagrams and diagnostic concepts, they struggled with direct image diagnosis. This limitation likely stems from insufficient exposure to specialized medical images during training, as general-purpose LLMs are not specifically trained on cytogenetic datasets. Successful karyotype analysis requires recognition of subtle morphological features and banding patterns that may not be adequately represented in their training data. Furthermore, the resolution and quality of training images may vary significantly from clinical standards. Future improvements in this domain would require specialized training on high-resolution, standardized karyotype images with consistent formatting and annotation protocols.

It is worth noting that specialized AI models have shown high accuracy in certain medical imaging tasks, such as the diagnosis of cervical cancer pathology and the detection of esophageal cancer in endoscopy [19,20]. However, these applications primarily function as support tools. Thus, addressing potential risks, ensuring safety and efficacy, and considering ethical responsibilities remain essential for AI’s broader application to health care [21]. The strong performance on theoretical knowledge questions suggests significant potential for LLMs in embryology education, particularly for smaller fertility clinics that may lack experienced senior embryologists or comprehensive training programs. Embryologists typically learn through hands-on experience during routine work, but facilities with limited case volumes or staff may struggle to provide adequate educational opportunities. This perspective is supported by recent work demonstrating that LLMs can effectively generate high-quality educational content and classification datasets tailored to specific pedagogical contexts, such as classroom dialog categorization [22,23]. Practical implementation could include interactive question and answer sessions for certification exam preparation, standardized explanations of complex procedures, case-based learning scenarios, and 24/7 availability for concept clarification. Their ability to process and analyze complex medical information, coupled with their limitations in specialized tasks, such as image interpretation, suggests a promising but carefully defined role in medical education and clinical support. The rapid evolution of AI is transforming medical education from traditional knowledge transfer to distributed cognitive systems, which requires educators to acquire collaborative skills with AI tools beyond conventional expertise, fostering learners’ problem-solving abilities and ethical reasoning while establishing a new educational paradigm that integrates human creativity with AI’s computational capabilities [24-26].

This study highlights the potential of LLMs to improve medical education and support clinical decisions in reproductive medicine, while reinforcing the need for human expertise and ethical oversight as technology advances.

### Limitations

This study has several limitations that should be considered when interpreting the results. First, the rapidly evolving nature of LLM technology means that the models evaluated (as of August 2024) may already have been updated with improved capabilities. The complete failure of Claude in data processing tasks, for instance, may reflect version-specific limitations rather than fundamental constraints of the platform. Second, we intentionally used baseline prompts without optimization to assess out-of-the-box performance. While this approach provides a realistic assessment of accessibility for nontechnical users, it likely underestimates the models’ full potential. Prompt engineering techniques, such as few-shot learning or chain-of-thought prompting, could substantially improve accuracy and consistency, particularly for complex data analysis tasks. Institution-specific prompt templates tailored to each LLM’s characteristics could further enhance performance and reduce the quality assurance burden identified in our results. Third, the knowledge assessment component faced constraints in question availability. The proprietary nature of professional certification examinations in embryology limited our test set to 10 questions curated by our institutional experts. This small sample size and potential selection bias may not fully represent the breadth of knowledge required in clinical embryology. Fourth, the image-based diagnostic assessment was limited by technical factors. The karyotype images used (600×450 pixels) may not have provided sufficient resolution for accurate chromosome band identification. The absence of standardized regions of interest or zoom capabilities may have contributed to the variable performance across models. These technical constraints highlight the importance of image quality standardization when implementing AI tools for medical image analysis. Finally, while our study demonstrated significant time savings in data processing, we did not assess the long-term reliability or the risk of automation bias. The potential for hallucinations and plausible but incorrect outputs remains a critical concern that requires continuous monitoring and validation protocols. Health care providers must maintain vigilance and establish robust verification procedures before integrating these tools into clinical workflows.

### Conclusion

This study demonstrates both the potential and limitations of current LLMs in reproductive medicine applications. While these tools showed promise for accelerating routine data analysis and supporting medical education, their failure in image interpretation tasks highlights critical limitations for clinical use. For smaller fertility clinics, LLMs offer promising support for data processing and staff training, potentially enhancing operational efficiency without the need for specialized technical infrastructure. However, to use these technologies safely and effectively in real clinical settings, it remains essential to establish well-defined procedures to verify their reliability, define clear rules for appropriate use, and ensure constant human involvement in making medical decisions. Future research should focus on training AI models specifically for reproductive medicine and systematically evaluating their impact on real-world clinical outcomes.

## Abbreviations

AI: artificial intelligence
ART: assisted reproductive technology
LLM: large language model
